# Inactivation of *Salmonella* Typhimurium and *Listeria monocytogenes* on ham with nonthermal atmospheric pressure plasma

**DOI:** 10.1371/journal.pone.0197773

**Published:** 2018-05-24

**Authors:** Karolina Anna Lis, Annika Boulaaba, Sylvia Binder, Yangfang Li, Corinna Kehrenberg, Julia Louise Zimmermann, Günter Klein, Birte Ahlfeld

**Affiliations:** 1 Institute of Food Quality and Food Safety, University of Veterinary Medicine Hannover, Foundation, Hannover, Germany; 2 Terraplasma GmbH, Garching, Germany; University of Campinas, BRAZIL

## Abstract

The application of cold atmospheric pressure plasma (CAP) for decontamination of sliced ready-to-eat (RTE) meat products (in this case, rolled fillets of ham), inoculated with *Salmonella* (*S*.) Typhimurium and *Listeria (L*.*) monocytogenes* was investigated. Cold atmospheric plasma (CAP) is an ionised gas that includes highly reactive species and ozone, interacting with cell membranes and DNA of bacteria. The mode of action of CAPs includes penetration and disruption of the outer cell membrane or intracellular destruction of DNA located in the cytoplasm. Inoculated ham was treated for 10 and 20 min with CAP generated by a surface-micro-discharge-plasma source using cost-effective ambient air as working gas with different humidity levels of 45–50 and 90%. The chosen plasma modes had a peak-to-peak voltage of 6.4 or 10 kV and a frequency of 2 and 10 kHz. Under the tested conditions, the direct effectiveness of CAP on microbial inactivation was limited. Although all treated samples showed significant reductions in the microbial load subsequent to plasma treatment, the maximum inactivation of *S*. Typhimurium was 1.14 lg steps after 20 min of CAP-treatment (*p*<0.05), and *L*. *monocytogenes* was reduced by 1.02 lg steps (*p*<0.05) using high peak-to-peak voltage of 10 kV and a frequency of 2 kHz regardless of moisture content. However, effective inactivation was achieved by a combination of CAP-treatment and cold storage at 8°C ± 0.5°C for 7 and 14 days after packaging under sealed high nitrogen gas flush (70% N_2_, 30% CO_2_). Synergistic effects of CAP and cold storage for 14 days led to a clearer decrease in the microbial load of 1.84 lg steps for *S*. Typhimurium (*p*<0.05) and 2.55 lg steps for *L*. *monocytogenes* (*p*<0.05). In the case of *L*. *monocytogenes*, subsequent to CAP-treatment (10 kV, 2 kHz) and cold storage, microbial counts were predominantly below the detection limit. Measurement showed that after CAP-treatment, surface temperature of ham did not exceed the room temperature of 22°C ± 2°C. With the application of humidity levels of 45–50%, the colour distance ΔE increased in CAP treated samples due to a decrease in L* values. In conclusion, effectiveness of CAP-treatment was limited. However, the combination of CAP-treatment and cold storage of samples under modified-atmospheric-conditions up to 14 days could significantly reduce microorganisms on RTE ham. Further investigations are required to improve effectiveness of CAP-treatment.

## Introduction

Ready-to-Eat (RTE) products imply quick availability, a high level of freshness, good quality and constant nutritional values. Above all, consumers have trust in microbial harmlessness. However, contamination could occur anytime throughout processing procedures and might be amplified by a long shelf life of RTE products. RTE products are not heated by the consumer before consumption, so microbial safety has to be guaranteed in a different way.

This product category also includes the very popular pre-sliced raw sausage products, e.g., rolled fillets of ham.

It should be noted that the production process of slicing and packaging raw sausage is associated with a surface extension which is prone to a higher contamination rate [1]. Occurring in the environment, *Listeria monocytogenes* can be transferred to ham during the entire production process [2], while *Salmonella* spp. is mainly transmitted by infected employees. Salmonellosis, the second most common bacterial gastrointestinal disease in Germany [3], causes diarrhoea, headaches and abdominal pain and an economic burden of approximately EUR 3 billion per year [4, 5]. Symptoms of listeriosis include fever, diarrhoea and vomiting and YOPIS (young, old, pregnant and immunosuppressed people) are at risk of a severe progress of the disease [6, 7].

The European Food Safety Authority (EFSA) reported an increase in food-borne disease outbreaks in the last years, mainly caused by food-borne pathogens like enterohaemorrhagic *Escherichia (E*.*) coli* (EHEC) and *Listeria monocytogenes*, as well as *Salmonella* spp. and *Staphylococcus* spp. (European Food Safety Authority, 2013). The Robert-Koch-Institute reported an increase in outbreaks of listeriosis and, despite a slight decrease, a high number, that of almost 13.000 cases of salmonellosis in 2016 in Germany [3]. Usual decontamination methods for an efficient reduction of microbial loads of food products include a heat treatment, e.g. canning food. Thus is associated with changes in product structure, colour and taste. New food processing technologies contain the ohmic heating or other mild heating-processing technologies or high pressure processing [8]. This enables the production of foods with a high nutritional content and a microbial safety throughout their shelf-life due to a shorter treatment duration [9].

The use of cold atmospheric-pressure plasma (CAP), also called nonthermal plasma, may close this gap. Plasma is the fourth state of matter and is generated by applying an electrical field to a working-gas [10]. This produces many different plasma species such as ions, free electrons, as well as reactive radical species and ozone with anti-microbial properties [11]. The plasma gas includes species such as NO, NO_2_, N_2_O, CO, CO_2_ and H_2_O_2_.

Additionally, this technique works close to ambient air temperature and is appropriate for processing freshly cut products, milk and dairy products preserving their product quality [12, 13]. CAP was initially developed for sanitation of medical heat sensitive devices and is also used for therapy of chronically infected wounds [12, 14, 15].

Present CAP systems are not standardised and differ in construction and in the use of comparatively expensive inert gases or ambient air. Studies showed promising results in reduction of different bacteria on different food surfaces [16, 17]. However, the plasma devices used in these studies operated with noble gases and treatment is only possible for small surface areas.

In the present study the effects of a CAP application using low-cost ambient air as a working gas and a surface-micro-discharge-plasma source (SMD plasma source) for decontamination of larger areas were investigated.

The primary issue was an effective inactivation of two zoonotic pathogens through CAP-treatment using varying plasma-modes and modifying humidity levels. The microbiological product stability was also checked during subsequent storage.

## Materials and methods

### Circular plasma system

**Fig 1:**
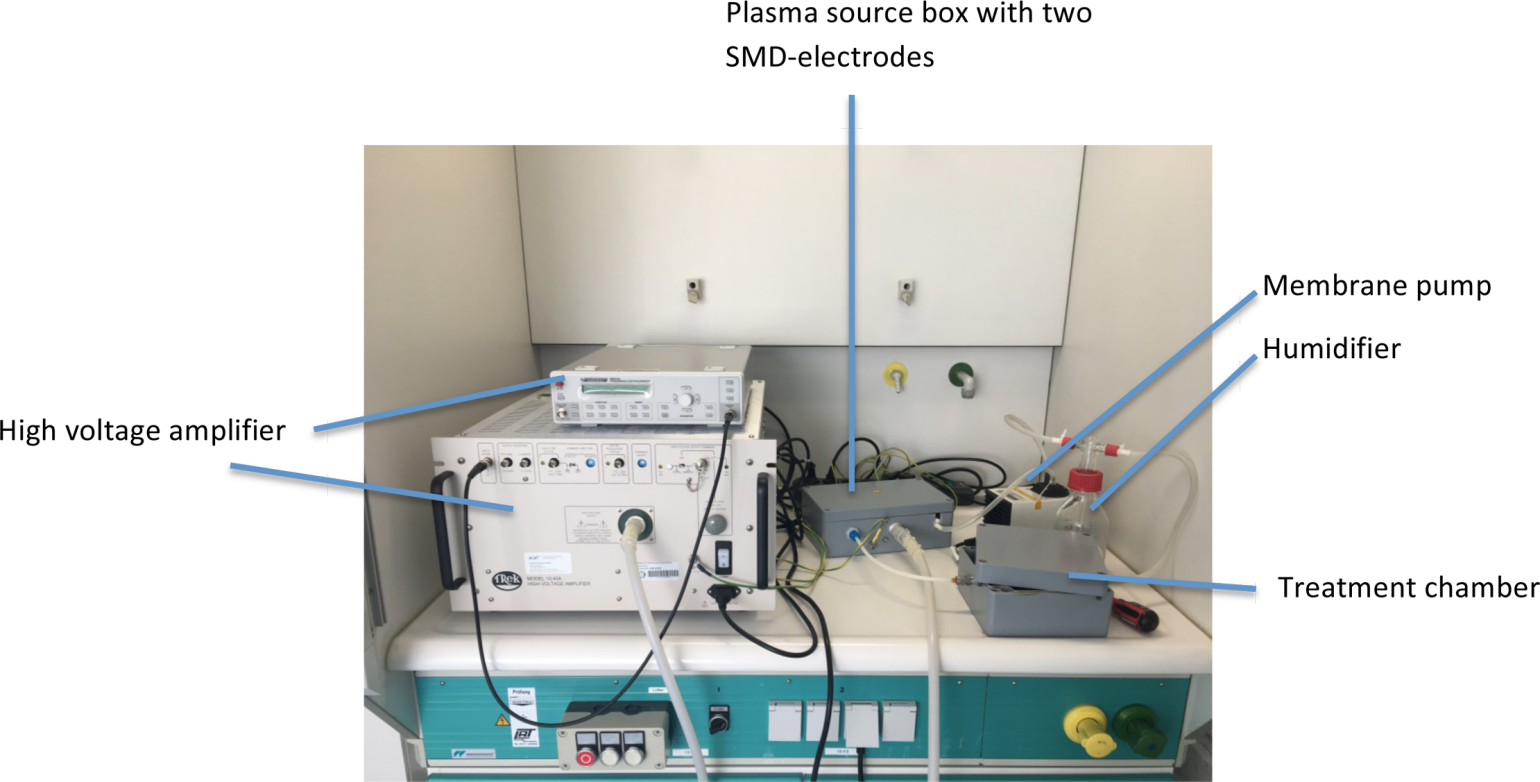
Circular plasma system.

The experimental set-up of this circular plasma system (Fig 1) consisted of two surface-micro-discharge-plasma sources with a coaxial structure (terraplasma GmbH, Garching, Germany). The terraplasma GmbH defined the SMD plasma source with a length of 10 cm and a diameter of 1 cm as a stainless steel mesh electrode with a spring shape covered by a quartz tube (electrical insulator) with a thickness of one mm surrounded by a copper tube-high voltage electrode. The plasma activated gas is produced inside the two cylindrical SMD-plasma sources embedded in the plasmabox by applying high voltage using a high voltage amplifier (model 10/40A, TREK Inc., USA) and cycled to the treatment chamber without a gas exchange with the surrounding air (closed circular system). Further components of this experimental set-up are a humidifier for moisturising of the carrier gas and a membrane pump (KNF, Freiburg Germany) with five standard litres per minute (slm) for a steady gas circulation.

In the experimental setup, two operating plasma modes were chosen to provide different gas phase chemistries with different mixing ratio of reactive oxygen species (ROS) and reactive nitrogen species (RNS). For each plasma modus the humidity concentration of feed gas was varied by either moisturising the ambient air up to 90% (‘wet’) or by using the natural air humidity (‘dry’) to investigate the influence of different humidity levels on the effectiveness of inactivation of both bacterial species on rolled fillets of ham (Table 1). The plasma activated gas was pumped from the plasmabox to the treatment chamber with a volume of about 2.6 L.

**Table 1:** Plasma operating conditions.

Plasma mode	Air humidity [%]	Input voltage (Vpp) [kV]	Frequency [kHz]	Power
1 *(wet)*	90	6.4	10	low
2 *(dry)*	45–50	6.4	10	low
3 *(wet)*	90	10	2	high
4 *(dry)*	45–50	10	2	high

During transportation of generated plasma species from the SMD-plasma source to the sample, highly reactive plasma-species induce further chemical reactions. Due to the ‘second’ collisions with air and/or water molecules further radical species are formed or decayed and are not able to reach the sample surface [18].

Depending on power operating conditions and changes in humidity different plasma chemistry is produced.

### Bacteria used for the experiments and culturing

Among the most common food-borne bacteria causing diseases in human are the Gram-negative and the Gram-positive pathogens *S*. *enterica* serovar Typhimurium (DSM 14221) and *L*. *monocytogenes* (field strain), respectively.

For each trial the bacterial strains were cultivated in Brain Heart Infusion broth (BHI) (Merck KGaA, Darmstadt, Germany) and incubated at 37°C for 24 hours (h). In a second step, 0.1 mL of this broth was inoculated in fresh BHI broth and incubated at 37°C for 24 h to achieve a bacterial concentration of approximately 10^8^ CFU/mL. BHI suspensions containing *S*. Typhimurium or *L*. *monocytogenes* were subsequently diluted to a final concentration of 10^7^ CFU/mL for *S*. Typhimurium and 10^5^ CFU/mL for *L*. *monocytogenes* depending on the infection dose for humans as published by EFSA in 2015 [19].

For colony counting, 0.1 mL of aliquots of a serial dilution (10^−2^ to 10^−6^) were plated on the surface of agar plates and incubated in accordance with ISO 6579 and ISO 11290–1. The enumeration of *S*. Typhimurium and *L*. *monocytogenes* were conducted using selective Xylose-Lysin-Desoxycholat agar (XLD) and Listeria-Brilliance agar (both Oxoid GmbH, Wesel, Germany), respectively. On XLD agar all transparent *S*. Typhimurium colonies with a black centre and a diameter of 1–2 mm were taken into account [20]. On Listeria-Brilliance agar all light blue to green *L*. *monocytogenes* colonies with an opaque zone and a diameter of 2 mm were counted [21]. Confirmations of these bacterial strains were conducted in previous experiments.

### In-vitro testing of agar plates and determination of plasma treatment times

Preliminary in vitro tests were carried out on XLD and Listeria-Brilliance agar plates to determine the plasma treatment times for rolled fillets of ham using the wet low-power mode. A volume of 0.1 mL of the *Salmonella*- and *Listeria* suspensions containing each approximately 10^7^ CFU/ml were plated on agar plates, air dried and plasma treated for 5, 10, 15 or 20 min, respectively. Afterwards, the control samples as well as the plasma-treated samples were incubated for 24 h (*S*. Typhimurium) and for 48h at 37°C (*L*. *monocytogenes*).

### Sampling, inoculation and treatment of rolled fillets of ham

Prior to the start of the experiment, sliced and packaged rolled fillets of ham of the type of an air dried sausage (Gut&Guenstig, Hamburg, Germany) had been purchased at a local grocery store (Foodservice Mios, Hannover, Germany) and stored in a commercial refrigerator at 8°C ± 0.5°C till start of the experiments. One batch of ham was used for a test run (batch number D-158XX) and one package contained 100 g of rolled fillets of ham. The sale denomination is low-fat (maximum 2% fat) cured rolled fillet of ham which is produced from *Musculus longissimus dorsi*.

Inoculation of the product surface was as follows: Three slices of rolled fillets of ham with a total weight of 25 g, in accordance with ISO 11290–1 and ISO 6579 formed a unit and were aseptically transferred to a plastic tray (227x178x30 mm, ES-Plastik, Hutthurm, Germany) [22, 23]. Shortly afterwards 0.1 mL of the inoculum was spread evenly on the surface. The period of time between inoculation and plasma treatment was for all samples less than two minutes. The amount of bacteria used for spiking ham fillets was 7.5x10^4^ CFU/g for *S*. Typhimurium and 6x10^3^ CFU/g for *L*. *monocytogenes* which are required for bacterial concentrations to cause clinical infections in humans.

For direct plasma application all samples were moved to the treatment-box and were CAP-treated for 0, 10 and 20 minutes, respectively; in contrast, non-treated samples were included as controls. The first group of samples was microbiologically analysed directly after plasma treatment, while the second and third group being sealed under high nitrogen gas flush (70% N_2_, 30% CO_2_) and stored for 7 and 14 days at 8°C ± 0.5°C.

The experiments were repeated as follows: For mode I 15 times, for mode II 10 times, for mode III and IV 6 times. A fresh bacterial broth was used for each run.

### Microbiological analysis

All microbiological examinations were performed directly after plasma treatment, in addition, and on storage days 7 and 14, in accordance with ISO-6579 und ISO-11290-1. One sample-unit with a weight of approximately 25 g was transferred to a stomacher bag (Grade Blender Bags Standard 400, Leicestershire, England), filled up with 225 mL of buffered peptone water (Oxoid GmbH, Wesel, Germany) and homogenised (200 rpm/2min) for 2 min. Serial dilutions were used for determining bacterial counts of *S*. Typhimurium, whereas suspensions of *L*. *monocytogenes* were stored at room temperature for one hour prior to enumeration. One millilitre of the diluted suspension was spread in triplicate (0.3, 0.3 and 0.4mL) on selective agar plates in accordance with the poured plate method and incubated for 24h or 48h at 37°C. The microbial counts were expressed as lg colony forming units per gramme ham (lg CFU/g). Microbial counts below the detection limit were integrated in the calculation with 5 CFU/mL.

All following inactivation rates were represented as lg difference between the initial microbial count and the microbial count after CAP-treatment or after CAP-treatment and storage for 7 or 14 days, using the formula (1):

$$lgDIF=\mathrm{lg}(N_{0}initial\;count-N_{t}\;post\;treatment\;(and\;storage)).$$ (1)

### Temperature measurements

Measurements of the surface temperature of ham were carried out before and after plasma treatment with a food infrared thermometer (BP 5F Food-Thermometer, Trotec, Aachen, Germany). The temperature measurement was carried out not later than one minute, after taking the treated samples out of the treatment chamber. Each temperature measurement was repeated three times.

### Surface colour

To monitor changes in colour, every sample of rolled fillets of ham was investigated before and after CAP-treatment using the colorimeter Minolta CR 400^®^ (Konica-Minolta GmbH, Langenhagen, Germany) on day 0 and after days 7 and 14 of MAP storage. Mean values were calculated from six internal measurements in accordance with the L*a*b* system of the CIE (Commission Internationale de l`Éclairage). The a*- (green to red) and b*- (blue to yellow) values are at one level and perpendicular to the L*-value (dark to light). Total colour distance ΔE was determined using the formula below (2). *Delta* (Δ) E described the difference of L*a*b*-measurements before and after the CAP-treatment. Delta-E indicates an objective difference between two colours, since it takes into account the difference between L*-, a*- and b*-values before and after treatment. Each colour measurement was repeated at six locations on the surface of the ham using the formula (2):

$$\Delta E=\sqrt{{\left(\Delta L*\right)}^{2}+{\left(\Delta a*\right)}^{2}{+\;(\Delta b*)}^{2}}$$ (2)

Changes in surface colour were also described with Hue (H) and Chroma (C), which can be calculated using the following formulas (3) and (4):

$$H=\arctan\left(\frac{b*}{a*}\right)$$ (3)

$$C=\sqrt{{a*}^{2}+{b*}^{2}}$$ (4)

### Statistical analysis

Data were processed with Excel® (MicrosoftTM) and analysed statistically using SAS Enterprise Guide 7.1. (SAS Institute Inc., Cary, North Carolina, USA). Microbial values were transferred to lg values. To evaluate normal distributions of the residues, PROC UNIVARIATE was used. A one-way analysis of variance (ANOVA) was performed followed by an REGWQ to compare experimental groups with control groups and each against one another. Furthermore, repeated measurements were analyzed by one way-ANOVA followed by the TUKEY multiple comparison test to indicate alterations during the storage period. The treatment (variants) and time (storage days) were the fixed terms in the model. A level of *p*<0.05 was considered significant. All treatment values were presented as means ± SD.

The non-normally distributed colour values were calculated using the nonparametric Kruskal-Wallis test. *P* values lower than 0.05 were noted as significantly different.

## Results and discussion

### In-vitro inactivation on agar plates

Preliminary in-vitro tests were carried out to examine the most effective plasma treatment times of plasma-mode I (6.4 kV, 10 kHz, wet). The maximum inactivation on agar plates of *S*. Typhimurium was 5.3 ± 0.2 lg steps after 10 min- and 5.7 ± 0.1 lg steps after 20 min of plasma treatment. The inactivation proved to be more effective than the inactivation of *L*. *monocytogenes* with 4.2 ± 0.2 lg steps after 10 min and 4.4 ± 0.3 lg steps after 20 min. In summary, inactivation of both bacterial species resulted in microbial counts on agar plates below the detection limit (5 CFU/mL) after 20 min of CAP-treatment.

Similar results were reported by Lee et al., who achieved reductions in *L*. *monocytogenes* between 0.9 and 7.6 lg steps after a two minute treatment using an atmospheric pressure plasma jet with different percentages of inert gases (He, N_2_ and O_2_) [24]. The mix of O_2_ and N_2_ achieved the highest levels of inactivation in this previous study. In contrast, in our study, only ambient air was used and treated not only a small surface area but also the whole agar plate.

Similar to our experiment, an inactivation of *Staphylococcus (S*.*) aureus* resulted in bacterial counts near or below the detection limit was shown by a 10 min CAP-treatment using a barrier-discharge plasma and ambient air as working gas [25].

A further study showed clearly shorter treatment times for inactivation of *E*.*coli* on agar plates with a radio-frequency (rf)–plasma using noble gas helium [26]. In summary, literature about studies at an early stage shows varying results in inactivation of bacteria in dependence of carrier gas and plasma source. In addition, the selected bacteria species may influence inactivation of different plasma modes [27].

### Effect of power on inactivation

**Fig 2:**
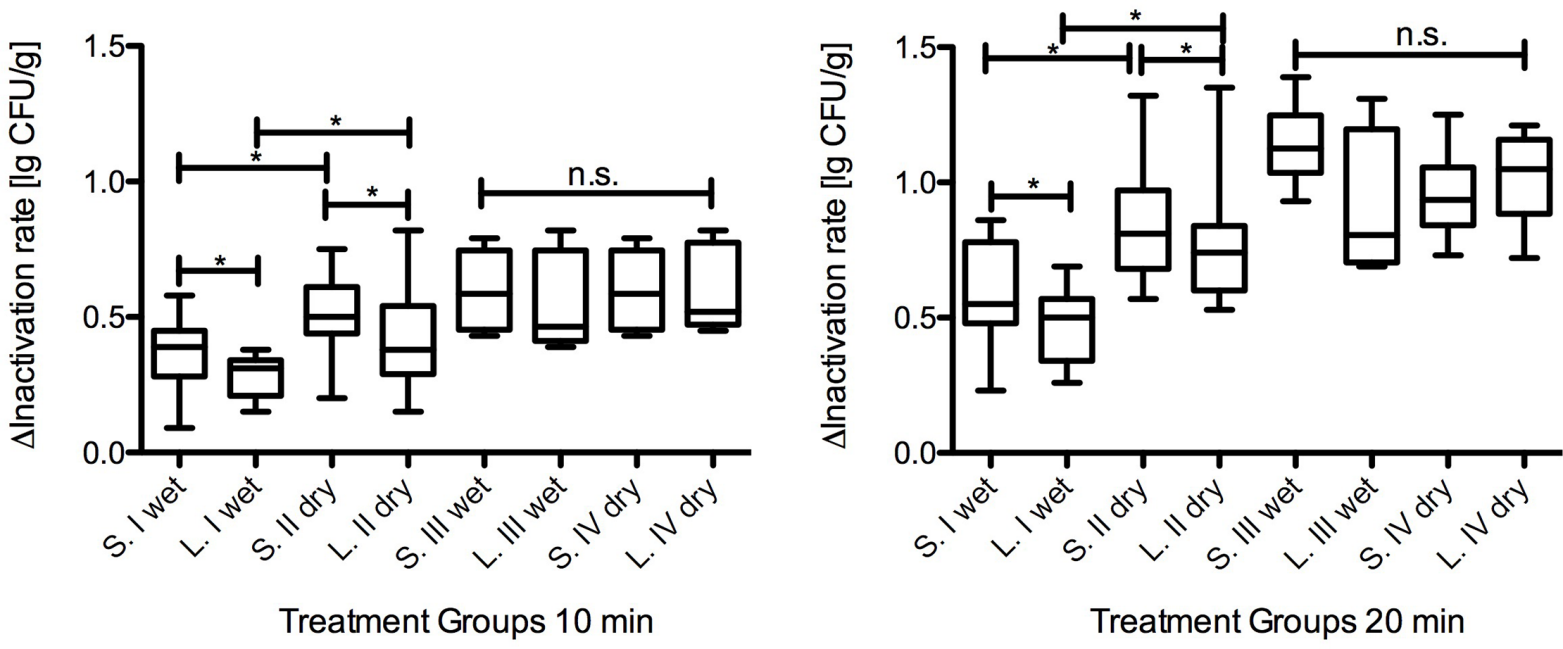
Inactivation of *S*. Typhimurium and *L*. *monocytogenes* after 10 and 20 min plasma treatment using four different plasma-modes. This figure shows inactivation of *S*. Typhimurium and *L*. *monocytogenes* after 10 min and 20 min CAP-treatment. Numbers I-IV are the four different trial groups. Represented significances are for groups I and II, and for groups III and IV, respectively. Results represent the mean ± standard error, *p* is defined as *p* > 0.05 *, no significant difference as n.s.

Our results showed that with increasing power of the plasma source a significantly higher bacterial inactivation was achieved for both *S*. Typhimurium and *L*. *monocytogenes* on the ham surface. Both high-power modes (III and IV) showed a significantly higher inactivation of bacteria compared to low-power modes (I and II). For example after 20 min CAP-treatment, the reduction in *S*. Typhimurium increased significantly from 0.59 ± 0.17 to 1.14 ± 0.15 lg steps using low- (I) or rather high-power (III). Reduction in *L*. *monocytogenes* also increased significantly from 0.46 (I) to 0.91 (III) lg steps due to the application of high-power after 20 min CAP-treatment (Fig 2).

In accordance with this study, Bauer et al. described also an increased inactivation of *S*. *aureus*, *L*. *monocytogenes* and two different *E*. *coli* strains when increasing power from 8.16 kV to 9.44 kV using ambient air as working gas and a DBD-plasma [28]. Winter et al. used a DBD-plasma and argon as carrier gas and also showed that inhibition of bacterial growth increased with higher discharge power of 5 W instead of 0.9 W [29].

Generated reactive species were the result of different collisions of produced electrons, ions, ROS, and RNS with different components of ambient air after applying an electric field to the SMD-source. Most of the plasma species were ROS and RNS, like atomic oxygen, O_3_, OH, NO and NO_2_ [11]. These might react with components of the bacterial outer cell membrane or diffuse through the cell wall into the cytoplasm and damage cell organelles as well as DNA [30].

The same authors also showed that with increasing power conditions ozone concentration decreases, whereas the content of more reactive plasma-species like N_2_O, NO_2_ and N_2_O_2_ increases [28, 29].

Through the use of noble gases, in some experiments higher bacterial inactivation was achieved than in our experimental mode [24, 31]. This is maybe due to a higher content of high-energy reactive plasma-species, above all, high-energy ions.

Nonetheless, the use of ambient air has the advantage over noble gas that it does not represent a finite source. Therefore, it is a cost-effective alternative, and more suitable for industrial applications.

### Impact of treatment time

The exposure-time to reactive plasma species also influenced the level of bacterial inactivation. Both the low- and high-power mode showed a clear time-dependent significant increase in bacterial inactivation by doubling the treatment time from 10 to 20 minutes for *S*. Typhimurium and for L. *monocytogenes* (Fig 2).

In agreement with Liao et al., a longer treatment time resulted in a longer exposure time to ROS and RNS and a higher accumulation of reactive plasma species. This resulted in higher inactivation rates after 20 min of CAP-treatment [32]. Studies using other food surfaces for CAP-treatment showed significant reductions in cell viability by increasing treatment time on freshly cut melons [33].

### Influence of humidity on reactive species

In addition to power, moisture content of working gas had an influence on inactivation of bacteria. The dry low power mode (II) showed a significantly higher inactivation of *S*. Typhimurium and *L*. *monocytogenes* than the wet low-power mode (I) (Tables 2–5).

**Table 2:** Results of the effects of humidification of air and different plasma modes on inactivation of *S*. Typhimurium.

Plasma mode	1 (wet)6.4 kV, 10 kHz	2 (dry)6.4 kV, 10 kHz	3 (wet)10 kV, 2 kHz	4 (dry)10 kV, 2 kHz
10 min/ 0 days storage	0.37±0.13	0.51±0.14	0.60±0.15	0.68±0.24
20 min/ 0 days storage	0.59±0.17	0.85±0.21	1.14±0.15	0.95±0.17
0 min/ 7 days storage	-0.08±0.2	0.007±0.14	0.11±0.12	-0.05±0.14
10 min/ 7 days storage	0.52±0.22	0.91±0.21	0.87±0.13	0.81±0.03
20 min/ 7 days storage	0.90±0.26	1.05±0.23	1.27±0.19	1.25±0.16
0 min/ 14 days storage	0.05±0.17	0.2±0.18	0.08±0.12	0.11±0.05
10 min/ 14 days storage	0.72±0.22	0.88±0.18	1.20±0.26	1.17±0.30
20 min/ 14 days storage	0.87±0.25	1.25±0.19	1.84±0.13	1.57±0.49

Results are presented as *lgDIF* = lg(*N initial count* – *N post treatment* (*and storage*)).

**Table 3:** Analysis of the effects of humidification of air and different plasma modes on inactivation of *S*. Typhimurium.

Significant differences between following groups:	1 and 2	3 and 4	1 and 3	2 and 4	1 and 4	2 and 4
10 min/ 0 days storage	n.s.	n.s.	n.s.	n.s.	n.s.	n.s.
20 min/ 0 days storage	*	n.s.	*	n.s.	*	n.s.
0 min/ 7 days storage	n.s.	n.s.	n.s.	n.s.	n.s.	n.s.
10 min/ 7 days storage	n.s.	n.s.	*	n.s.	*	n.s.
20 min/ 7 days storage	n.s.	n.s.	*	n.s.	*	n.s.
0 min/ 14 days storage	n.s.	*	n.s.	n.s.	*	n.s.
10 min/ 14 days storage	n.s.	n.s.	*	*	*	*
20 min/ 14 days storage	*	n.s.	*	*	*	*

Significance is defined as *p*<0.05 *. n.s. = no significance. The groups 0 min/7 days storage and 0 min/14 days storage were negative controls.

**Table 4:** Results of the effects of humidification of air and different plasma modes on inactivation of *L*. *monocytogenes*.

Plasma mode	1 (wet)6.4 kV, 10 kHz	2 (dry)6.4 kV, 10 kHz	3 (wet)10 kV, 2 kHz	4 (dry)10 kV, 2 kHz
10 min/ 0 days storage	0.28±0.07	0.33±0.11	0.55±0.18	0.59±0.16
20 min/ 0 days storage	0.46±0.13	0.69±0.13	0.91±0.18	1.02±0.18
0 min/ 7 days storage	0.11±0.1	0.24±0.28	0.06±0.1	0.07±0.12
10 min/ 7 days storage	0.46±0.1	0.52±0.15	1.03±0.53	0.92±0.28
20 min/ 7 days storage	0.65±0.14	0.78±0.28	1.37±0.66	1.60±0.1
0 min/ 14 days storage	0.06±0.07	0.33±0.11	0.06±0.09	0.09±0.07
10 min/ 14 days storage	0.51±0.17	1.08±0.51	1.84±0.2	1.60±0.08
20 min/ 14 days storage	0.81±0.3	1.68±0.70	2.35±0.59	2.55±0.67

Results are presented as *lgDIF* = lg(*N initial count* – *N post treatment* (*and storage*)).

**Table 5:** Analysis of the effects of humidification of air and different plasma modes on inactivation of *L*. *monocytogenes*.

Significant differences between following groups:	1 and 2	3 and 4	1 and 3	2 and 4	1 and 4	2 and 4
10 min/ 0 days storage	n.s.	n.s.	*	*	*	*
20 min/ 0 days storage	*	n.s.	*	*	*	*
0 min/ 7 days storage	n.s.	n.s.	n.s.	n.s.	n.s.	n.s.
10 min/ 7 days storage	n.s.	n.s.	*	*	*	*
20 min/ 7 days storage	n.s.	n.s.	*	*	*	*
0 min/ 14 days storage	n.s.	n.s.	n.s.	n.s.	n.s.	n.s.
10 min/ 14 days storage	*	n.s.	*	*	*	*
20 min/ 14 days storage	*	n.s.	*	*	*	*

Significance is defined as *p*<0.05 *. n.s. = no significance. The groups 0 min/7 days storage and 0 min/14 days storage were negative controls.

Other studies also described a correlation between inactivation rates and gas humidity. A higher reduction in *Bacillus* (*B*.) *atrophaeus* spores was achieved by increasing humidity-levels by up to 50% [34]. Muranyi et al. reported a higher inactivation of *Aspergillus* (*A*.) *niger* with increasing relative gas humidity of up to 80% using synthetic air, as well [35].

In the present study we hypothesised that in both low-power modes (I and II) primarily the bactericidal working ROS and ozone were the major factor for inactivation of both bacterial species. Ozone is known for its oxidative stress and potential to exceed the ability of detoxification and repair of cells [36]. In addition to ozone, highly reactive hydroxyl radicals (OH^-^) are also formed [37]. These radicals have a high bactericidal effect. Although the lifetime of these OH^-^ radicals is longer with increasing humidity the influence of OH^-^ radicals on *S*. Typhimurium and *L*. *monocytogenes* is limited due to the distance from the plasma source to the surface of ham.

Some authors reported that the ozone concentration decreases with increasing humidity. Thus, moisture-dependent quenching effects of ozone with water molecules in the surrounding air or with moisture of the surface of ham or a low ozone susceptibility of both bacteria species could explain the low inactivation of bacteria [36, 38]. The thin water film around the micro-organism cells might also protect them against plasma species [39].

This would explain a lower inactivation of bacteria in the wet low-power plasma mode (I).

In contrast, both high-power modes (III and IV) showed no significant differences between wet or dry mode (Tables 2–5). Shimizu et al. described that by applying high power (III and IV) the fraction of the highly bactericidal acting excited nitrogen species increases [40]. Furthermore, these excited reactive nitrogen species are able to quench the ozone in a further chemical reaction. Both the formed RNS and nitric oxide (NO) might have a higher bactericidal effect than ozone and the reactive oxygen species. If the high power-mode with 10 kV yields ozone quenching, this might be responsible for the higher bacterial reduction. In accordance with our results, previous studies with plasma jets showed a positive influence on the longevity of reactive plasma species although ozone concentration decreases with increased humidity [36, 41].

In summary, it might be assumed that the negative influence of moisture on the ozone concentration was compensated by a higher variety and a longer half-life of RNS as high reactive plasma species in high-power modes (III and IV). Nevertheless, further investigations are necessary to understand the interaction of the plasma species with the bacteria on the surface of ham.

### Microbial inactivation

All experiments were conducted under conditions previously identified as the most appropriate ones for inactivation on agar plates.

Bacterial delta-inactivation-rate of low-power modes (I and II) resulted in significant higher reductions in *S*. Typhimurium than in *L*. *monocytogenes* (Fig 2). In contrast, experiments conducted with high-power modes III and IV showed no difference in inactivation between *S*. Typhimurium and *L*. *monocytogenes*. These settings also showed significantly higher germ inactivation than low-power modes (Fig 2).

As a high amount of ROS and ozone were postulated to be responsible for bacterial reduction in the low-power but not in the high-power modes, we assumed a different susceptibility of *S*. Typhimurium and *L*. *monocytogenes* against ROS and ozone. As in both high-power modes a higher bacterial inactivation was achieved due to the generation of excited reactive nitrogen species in addition to ozone. It seems that there is no difference in the susceptibility of both pathogens against RNS and a stronger effect of these reactive species can be assumed.

However, it can be presumed that differences in the cell walls between Gram-positive and Gram-negative bacteria might have an influence on their resistance against external stress such as heat, drought, pressure and possibly cold plasma. Some authors hypothesise that effectiveness of CAP-treatment might depend on the thickness of this cell membrane [42, 43].

The literature describes two main mechanisms of cell inactivation and destruction by reactive plasma species: Plasma species can either react directly with the outer cell membrane, or they diffuse through the lipid bilayer into the cytoplasm and damage cell organelles and DNA by destroying covalent bonds and triggering chemical reactions [44].

The former inactivation mechanism is reported by Maksudbek et al. who describes a high effectiveness of ROS and RNS against the cell membrane components of Gram-negative bacteria lipopolysaccharides and peptidoglycans [45]. In addition, a cell breakage of Gram-negative bacteria occurs by lipid peroxidation of lipopolysaccharides [27].

In Gram-positive bacteria, ROS and RNS diffused through bacterial cell membranes or were transported by ion channels and reacted with intracellular organelles and resulted in cell shrinkage [46, 47]. In the low-power modes (I and II), ROS and ozone were considered to be a major component for the inactivation of bacteria. In this experimental mode, *S*. Typhimurium was significantly more highly inactivated than *L*. *monocytogenes* (Fig 2). Ozone as an example for reactive oxygen species interacts mainly with the cell membrane of bacteria and can primarily destroy the thinner cell membrane of the Gram-negative pathogen *S*. Typhimurium by oxidation reactions [48, 49].

Using high-power modes the concentration of excited high bactericidal nitrogen increases, these particles could interact with the cell membrane, but they could also enter the cell interior and destroy cell organelles [50]. As a consequence, the bacterial cells can be destroyed regardless of their cell wall thickness.Previous studies on application of CAP-treatment to food surfaces use low-power modes are in good accordance with the results in this study using low-power modes. Ziuzina et al. reported a higher susceptibility of *S*. Typhimurium than of *L*. *monocytogenes* to CAP-treatment after inoculation of food surfaces, such as tomatoes and strawberries [42]. In addition, the study by Critzer et al. also confirmed that *E*. *coli* were primarily damaged by cell leakage and to a lesser extent also by DNA damage to the reactive plasma-species [43]. Further studies showed an inactivation of *S*. *aureus* after accumulation of ROS in the intracellular space and cells were found to exhibit an almost intact cell membrane [30]. Through lipid oxidation, Gram-negative and -positive bacterial species are additionally exposed to oxidative stress, which can also lead to enzyme inactivation and DNA damage [46].

However, the amount of inoculated cells must also be taken into consideration. In this study, more inoculated *S*. Typhimurium was inactivated significantly higher than less inoculated *L*. *monocytogenes* by treatment with low-power mode (I and II). When using high-power mode (III and IV), inactivation of both bacterial species was approximately the same. These results might confirm the assumption that Gram-positive *L*. *monocytogenes* are more resistant to low-power CAP-treatment due to their bacterial cell membrane characteristics.

As mentioned above, an approximately 1-lg step higher cell concentration was chosen for *S*. Typhimurium. Some authors described a clear dependence of the initial count of inoculated bacteria and effectiveness of germ inactivation in relation to the treatment time [51, 52].

Inactivation was higher for *S*. Typhimurium (initial bacterial load ~7,5x10^4^ CFU/g) than for *L*. *monocytogenes* (initial bacterial load ~6x10^3^ CFU/g) in both low-power modes, despite a higher inoculation dose for *S*. Typhimurium. High-power modes (III, IV) showed no significant differences.

Our results, which showed no clear correlation between initial bacterial load and inactivation using low- and high-power plasma-modes (I-IV), are in accordance with those of Ziuzina et al., who also found no correlation [42].

The formation of a biofilm prior to plasma treatment can be ruled out due to the short time period between inoculation and treatment.

### Influence of surface structure

In contrast to our experiments using agar plates, where a germ reduction was achieved for levels close or below the detection limit, inactivation rates on ham were limited. Thus, the effectiveness of plasma treatment decreased when applying CAP to the more complex surface of ham. Therefore, it is obvious that composition of the matrix and surface topography play important roles in inactivation when using gas plasmas.

Surface structure of the treated matrix may also influence the viability of *S*. Typhimurium and *L*. *monocytogenes* cells. A previous study obtained different D-values, defined as exposure time required to reduce the germ population one lg or 90%, for various Gram-negative (for example, *E*. *coli*) and Gram-positive (for example, *Bacillus* spp.) bacteria on different surfaces, whereas the lowest D-value was achieved for polypropylene and the highest for paper [53]. The lower inactivation on ham might be the result of the rough and uneven structure of the surface. Many niches and cavities protect bacterial cells from reactive plasma species. Moreover, it is assumed that an active migration of microorganisms from the surface into the interior of the tissue is possible [54, 55]. Thereby, the speed of migration, called internalisation, can depend on several factors, such as bacterial cell size (small bacteria can migrate faster than large ones), the occurrence of virulence factors like flagellae of *Salmonella* spp., as well as the texture and pore size of the plasma-treated matrix. Perni et al. also discovered that there is a variation in migration rates depending on the bacterial species (e.g. 300 μm min^-1^ for *E*. *coli*), the surface properties and the water content [54].

It has also been described that bacteria can be drawn into deeper tissue layers by capillary forces [56]. As the surface of ham is naturally slightly moist and bacteria were inoculated in a moist broth, this effect cannot be ruled out.

### Influence of temperature

The influence of temperature of plasma gas and its impact on inactivation of bacterial cells has to be considered, too. In order to rule out an influence of heat of the plasma gas on inactivation of bacteria on the surface of ham, continuous temperature measurements of ham surface were carried out before and after plasma treatment (data not shown). According to Fröhling et al., the measured temperature never exceeded 30°C, so that germ inactivation due to the effect of temperature can be ruled out [57].

### Posttreatment storage

Reactive plasma species damaged the bacterial cell-envelope and accumulated in the intracellular room as described above.

Since bacterial cells were transferred to a nutrient broth directly after CAP-treatment, potentially sublethal damaged cells, defined as non-lethal injured cells, could revitalise or repair mechanisms of the bacterial cell could compensate cell damage [58]. Sublethal damaged cells showed intact enzyme activity, metabolism and cellular respiration, but no growth on agar and they are more susceptible to environmental influence [58, 59].

We therefore investigated bacterial inactivation depending on the duration of storage. The aim was to verify whether a reduction in the number of cells was achieved during storage, thereby demonstrating the detection of sublethal damaged bacterial cells by CAP-treatment.

**Fig 3:**
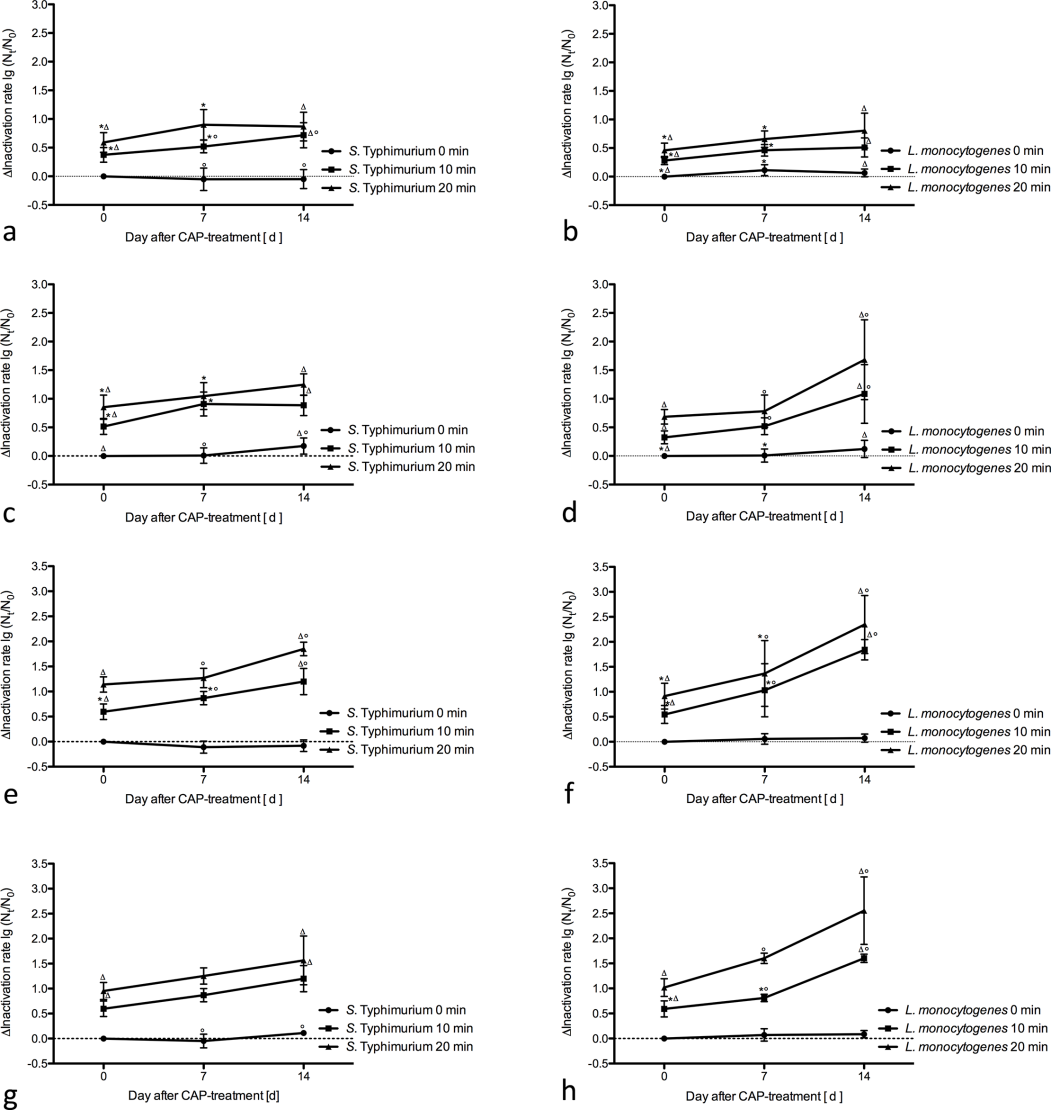
Inactivation of *S*. Typhimurium and *L*. *monocytogenes* during storage. Results represent the ± standard error in lg CFU/g as a difference to the control group. Same symbols indicate significant differences between control group and plasma treated and stored sample for one treatment time. Significance is defined as *p*<0.05. The groups ‘*S*. Typhimurium 0 min’ and ‘*L*. *monocytogenes* 0 min’ were negative controls. * = significant difference between day 0 and 7. Δ = significant difference between day 0 and 14.° = significant difference between day 7 and 14. a = I. *S*. Typhimurium (6.4 kV, 10 kHz, wet). b = I. *L*. *monocytogenes* (6.4 kV, 10 kHz, wet). c = II. *S*. Typhimurium (6.4 kV, 10 kHz, dry). d = II. *L*. *monocytogenes* (6.4 kV, 10 kHz, dry). e = III. *S*. Typhimurium (10 kV, 2 kHz, wet). f = III. *L*. *monocytogenes* (10 kV, 2 kHz, wet). g = IV. *S*. Typhimurium (10 kV, 2 kHz, dry). h = IV. *L*. *monocytogenes* (10 kV, 2 kHz, dry).

For this purpose, CAP-treated ham was packed under MAP-conditions und stored at 8°C ± 0.5°C. During 14 days of storage under MAP conditions, measurements were taken at days 7 and 14. The untreated control showed a slight increase in or a constant concentration of bacteria. As shown in Fig 3, a significantly additional inactivation-increase of bacterial cells was observed in all treated samples. Furthermore inactivation of bacteria during storage followed the same inactivation kinetics like after direct CAP-treatment.

This additional bacterial reduction can be seen as a surrogate parameter for sublethally damaged bacterial cells, as a delayed effect of plasma or a longer half-life of the ROS and RNS on the surface of ham at cooler ambient temperatures.

The death of sublethally-damaged bacterial cells is explained in the literature by different reaction-mechanisms of plasma-radicals with bacterial cell components. Han et al. described the decrease in cell concentration during storage as an effect of long-lived plasma species produced by indirect plasma treatment in [60]. Generated long-lived ROS interacted with the cell-envelope of *E*. *coli* during storage, while in the pathogen *S*. *aureus* an accumulation and destruction of DNA inside the cell was observed.

However, other mechanisms such as a temporally staggered breakdown of the membrane potential, or a destruction of the lipid-bilayer, ion channels and ion pumps might influence the cells during storage as well [61, 62].

These mechanisms could lead to an increased death of bacterial cells and further investigations are necessary to evaluate the effects of generated ROS and RNS on cell damage after treatments caused by our plasma system which operated with ambient air.

In summary, our results suggest the presence of sublethally damaged bacterial cells due to CAP-treatment, which survived initial treatment but got lost during storage.

However, with the high-power mode (III and IV) a higher initial inactivation shown and a significantly higher inactivation rates during storage were achieved for both bacterial species.

An additional inactivation during storage depending on the initial inactivation rate supports the assumption, that further inactivation is connected to mechanism initiated by plasma treatment. Besides a higher rate of immediate inactivated cells, high-power plasma-modes (III and IV) might have caused initial higher but still sublethal cell damage or damage affecting more bacteria cells, which led to a higher additional inactivation rate during storage.

While CAP showed a less effective reduction of *L*. *monocytogenes* than on *S*. Typhimurium, inactivation of *L*. *monocytogenes* was significantly higher than that of *S*. Typhimurium at days 7 and 14 during storage, especially in the dry high-power plasma-mode (IV). Reduction comprising up to 2.55 ± 0.67 lg steps was achieved during storage.

As described above, reactive plasma species of the high-power mode were effective against both bacterial species included in the study. Thus, we expected a higher inactivation rate when lower amounts of *L*. *monocytogenes* cells were inoculated. Nevertheless, a higher inactivation rate was detected only after storage. These results indicate a time-lagged effect and the presence of sublethally damaged cells. In conclusion, *L*. *monocytogenes* was more resistant against reactive plasma species but seem to be sublethally damaged.

Although a maximum inactivation of bacteria was only achieved after two weeks of storage, no bacterial growth could be detected at the beginning of storage. Thus, plasma treatment can at least effectively inhibit further bacterial growth on food surfaces during storage time.

### Colour measurements

Visual impressions of products play an important role in consumer acceptance. Above all, the colour of products is used to represent freshness and good quality [63].

Although one batch of ham was used for a test run, significant differences in colour were measurable within a batch prior to plasma treatment. Therefore, the measured L*-, a*- and b*-values were converted to Chroma and Hue, which belong to the LCH colour space (DIN 6174) and are identical to the CIE colour space (DIN 5033–2). The L-value (lightness) remains unchanged in both systems.

Delta E indicates an objective difference between two colours, since it takes into account the difference between L*-, a*- and b*-values before and after treatment. If this colour distance is known, an objective statement can be made as to how strongly two colours differ from each other regardless of varying initial colours [64]. Up to a value of delta E (Δ) < 2.5, no colour difference is noticed by the consumer [65]. Perceptible colour deviations can be perceived by untrained persons from delta E values above 2.5 [24, 65].

Differences in Hue and Chroma were observed for both untreated controls- and CAP-treated samples after direct CAP-treatment as well as after storage (Tables 6–8). In particular, samples treated with low-power showed significant colour deviations, which were particularly noticeable in the dry test groups. The high-power treatment showed a significant colour deviation in the samples inoculated with *S*. Typhimurium, predominantly in the dry plasma-mode.

**Table 6:** Colorimetric measurements of control and CAP-treated samples inoculated with *S*. Typhimurium.

**Plasma Mode I**				**Plasma Mode II**		
	Control group	Difference between control group and group after 10 min treatment	Difference between control group and group after 20 min treatment	Difference between first and second measurement of the control group	Difference between control group and group after 10 min treatment	Difference between control group and group after 20 min treatment
	Initial values at day 0			Initial values at day 0		
ΔE	0	2.60±0.50	2.66±0.49	0	1.84±0.95	2.53±0.70
	After 7 days of storage under MAP conditions			After 7 days of storage under MAP conditions		
ΔE	2.85±1.59	3.29±1.73	2.66±1.1	1.53±0.63	4.05±0.91	4.26±1.42
	After 14 days of storage under MAP conditions			After 14 days of storage under MAP conditions		
ΔE	2.24±1.22	2.77±1.47	2.39±1.12	1.85±0.75	3.19±1.69	3.64±1.49
**Plasma Mode III**				**Plasma Mode IV**		
	Control group	Difference between control group and group after 10 min treatment	Difference between control group and group after 20 min treatment	Difference between first and second measurement of the control group	Difference between control group and group after 10 min treatment	Difference between control group and group after 20 min treatment
	Initial values at day 0			Initial values at day 0		
ΔE	0	1.38±0.74	2.31±0.86	0	1.88±0.53	2.49±1.55
	After 7 days of storage under MAP conditions			After 7 days of storage under MAP conditions		
ΔE	2.21±1.47	3.40±0.98	3.19±1	1.51±0.39	4.40±0.47	3.50±1.44
	After 14 days of storage under MAP conditions			After 14 days of storage under MAP conditions		
ΔE	3.69±1.56	3.20±0.84	3.41±0.61	2.40±0.73	4.52±0.93	5.24±1.60

**Table 7:** Colorimetric measurements of control and CAP-treated samples inoculated with *L*. *monocytogenes*.

**Plasma Mode I**				**Plasma Mode II**		
	Control group	Difference between control group and group after 10 min treatment	Difference between control group and group after 20 min treatment	Difference between first and second measurement of the control group	Difference between control group and group after 10 min treatment	Difference between control group and group after 20 min treatment
	Initial values at day 0			Initial values at day 0		
ΔE	0	2.21±0.82	2.42±1.02	0	2.19±1.14	2.93±1.06
	After 7 days of storage under MAP conditions			After 7 days of storage under MAP conditions		
ΔE	1.50±0.57	3.75±0.87	3.49±1.28	1.76±0.91	4.13±1.05	4.33±0.91
	After 14 days of storage under MAP conditions			After 14 days of storage under MAP conditions		
ΔE	1.57±0.73	3.32±0.72	2.96±0.76	1.96±0.60	3.82±1.21	4.70±1.93
**Plasma Mode III**				**Plasma Mode IV**		
	Control group	Difference between control group and group after 10 min treatment	Difference between control group and group after 20 min treatment	Difference between first and second measurement of the control group	Difference between control group and group after 10 min treatment	Difference between control group and group after 20 min treatment
	Initial values at day 0			Initial values at day 0		
ΔE	0	2.08±0.28	2.23±0.36	0	2.91±1.50	3±1.44
	After 7 days of storage under MAP conditions			After 7 days of storage under MAP conditions		
ΔE	2.26±1.13	3.09±0.70	3.23±1.54	2.57±1.51	3.35±1.70	3.62±0.41
	After 14 days of storage under MAP conditions			After 14 days of storage under MAP conditions		
ΔE	2.04±0.94	4.15±0.88	3.25±1.38	2.57±1.22	3.97±0.86	4.55±1.56

**Table 8:** Overview of the results of analysis of delta E measurements.

	*S*. Typhimurium				*L*. *monocytogenes*			
	Plasma-mode 1	Plasma-mode 2	Plasma-mode 3	Plasma-mode 4	Plasma-mode 1	Plasma-mode 2	Plasma-mode 3	Plasma-mode 4
0 min/0 vs. 10min/0	*	n.s.	n.s.	n.s.	*	n.s.	n.s.	n.s.
0min/0 vs. 20min/0	*	*	n.s.	n.s.	*	*	n.s.	n.s.
0min/0 vs. 0min/7	*	n.s.	n.s.	n.s.	n.s.	n.s.	n.s.	n.s.
0min/0 vs. 10min/7	*	*	*	*	*	*	*	n.s.
0min/0 vs. 20min/7	*	*	*	*	*	*	*	*
0min/0 vs. 0min/14	*	n.s.	*	n.s.	n.s.	n.s.	n.s.	n.s.
0min/0 vs. 10min/14	*	*	*	*	*	*	*	*
0min/0 vs. 20min/14	*	*	*	*	*	*	*	*
10min/0 vs. 20min/0	n.s.	n.s.	n.s.	n.s.	n.s.	n.s.	n.s.	n.s.
10min/0 vs. 0min/7	n.s.	n.s.	n.s.	n.s.	n.s.	n.s.	n.s.	n.s.
10min/0 vs. 10min/7	n.s.	*	n.s.	n.s.	*	n.s.	n.s.	n.s.
10min/0 vs. 20min/7	n.s.	*	n.s.	n.s.	n.s.	n.s.	n.s.	n.s.
10min/0 vs. 0min/14	n.s.	n.s.	n.s.	n.s.	n.s.	n.s.	n.s.	n.s.
10min/0 vs. 10min/14	n.s.	n.s.	n.s.	n.s.	n.s.	n.s.	n.s.	n.s.
10min/0 vs. 20min/14	n.s.	*	n.s.	n.s.	n.s.	n.s.	n.s.	n.s.
20min/0 vs. 0min/7	n.s.	n.s.	n.s.	n.s.	n.s.	n.s.	n.s.	n.s.
20min/0 vs. 10min/7	n.s.	n.s.	n.s.	n.s.	n.s.	n.s.	n.s.	n.s.
20min/0 vs. 20min/7	n.s.	n.s.	n.s.	n.s.	n.s.	n.s.	n.s.	n.s.
20min/0 vs. 0min/14	n.s.	n.s.	n.s.	n.s.	n.s.	n.s.	n.s.	n.s.
20min/0 vs. 10min/14	n.s.	n.s.	n.s.	n.s.	n.s.	n.s.	n.s.	n.s.
20min/0 vs. 20min/14	n.s.	n.s.	n.s.	n.s.	n.s.	n.s.	n.s.	n.s.
0min/7 vs. 10min/7	n.s.	*	n.s.	n.s.	*	*	n.s.	n.s.
0min/7 vs. 20min/7	n.s.	*	n.s.	n.s.	*	*	n.s.	n.s.
0min/7 vs. 0min/14	n.s.	n.s.	n.s.	n.s.	n.s.	n.s.	n.s.	n.s.
0min/7 vs. 10min/14	n.s.	n.s.	n.s.	n.s.	*	n.s.	n.s.	n.s.
0min/7 vs. 20min/14	n.s.	*	n.s.	*	*	*	n.s.	n.s.
10min/7 vs. 20min/7	n.s.	n.s.	n.s.	n.s.	n.s.	n.s.	n.s.	n.s.
10min/7 vs. 0min/14	n.s.	*	n.s.	n.s.	*	n.s.	n.s.	n.s.
10min/7 vs. 10min/14	n.s.	n.s.	n.s.	n.s.	n.s.	n.s.	n.s.	n.s.
10min/7 vs. 20min/14	n.s.	n.s.	n.s.	n.s.	n.s.	n.s.	n.s.	n.s.
20min/7 vs. 0min/14	n.s.	*	n.s.	n.s.	*	*	n.s.	n.s.
20min/7 vs. 10min/14	n.s.	n.s.	n.s.	n.s.	n.s.	n.s.	n.s.	n.s.
20min/7 vs. 20min/14	n.s.	n.s.	n.s.	n.s.	n.s.	n.s.	n.s.	n.s.
0min/14 vs. 10min/14	n.s.	n.s.	n.s.	n.s.	*	n.s.	n.s.	n.s.
0min/14 vs. 20min/14	n.s.	*	n.s.	n.s.	n.s.	*	n.s.	n.s.
10min/14 vs. 20min/14	n.s.	n.s.	n.s.	n.s.	n.s.	n.s.	n.s.	n.s.

Significance is defined as *p*<0.05*. N.s. = no significance.

The increase in colour distance delta E exceeded the value of 3 by far for all dry treated samples. This increase could also be observed in the control group after storage, but was significantly higher in the dry CAP-treated samples.

As the colour variations also occured in the untreated control samples, they may result from repackaging of ham. Even measurement inaccuracies can lead to colour deviations because colour measurement was usually carried out at two different points on a slice of ham. Our results indicate that the significant increase in colour deviation in all dry CAP-treated groups is based on an evaporation of moisture of ham. The colour-measurement showed an increase in the a*- and L*-values in the most of dry CAP-treated samples, which suggest a redder-darkening of the surface of the ham (S1, S2, S3 and S4 Tables).

In contrast, in the wet CAP-treatment the ham kept its initial colour in the majority of samples. Since humidity was raised to 90% and this high humidity level led to lower moisture evaporation, no dehydration associated colour change occurs.

Consumer acceptance may not be affected by plasma treatment, since a redder colour of raw-ham products is highly accepted by the consumer. Opposed, greening and/or brightening of raw ham surfaces is may be associated by the consumer with microbial contamination or a not microbiologically safe product [64]. In our study no greening of the CAP-treated ham was observed, consumer acceptance can still be assumed.

Nevertheless, further sensory investigations like the hedonic-tests must be carried out with consumers (panelists) and with expert sensory assessors [66–68].

## Conclusion

The objective of this experimental study was to analyse inactivation of *S*. Typhimurium and *Listeria monocytogenes* on the surface of ham by CAP-treatment. Results should indicate whether CAP could be a suitable method for increasing food safety in ready-to-eat meat products.

Although the bacterial inactivation was limited whilst using low-power modes, the application of high-power modes resulted in a rather small, but significant reduction of bacteria by approximately 1 lg step.

The combination of high-power plasma mode and cold storage reduced *S*. Typhimurium by 1.85 lg steps, whereas *L*. *monocytogenes* was reduced by 2.55 lg steps near or below the detection limit. It should be noticed the easy integrity of using a cost effective ambient air system for food treatment. Moreover, the type of application conforms to the way it might be implemented and used in future food production. Also, post-treatment storage is compatible with many industrial processes and their further distribution.

Lastly, further tests should be carried out to test the effectiveness of plasma against further bacterial species and different food surfaces and to enhance the plasma power to achieve a complete bacterial reduction on food surfaces.

The method is most beneficial as there is no waste material to be disposed of and it is therefore environmentally friendly.

## Supporting information

S1 Table Cololorimetric measurements of control and CAP treated samples inoculated with *S*. *enterica* serovar Typhimurium. Results represent the mean ± the standard error for L*, a*, b* values and for Hue and Chroma.
(DOCX)

S2 Table Cololorimetric measurements of control and CAP treated samples inoculated with *S*. *enterica* serovar Typhimurium. Results represent the mean ± the standard error for L*, a*, b* values and for Hue and Chroma.
(DOCX)

S3 Table Cololorimetric measurements of control and CAP treated samples inoculated with *L*. *monocytogenes*. Results represent the mean ± the standard error for L*, a*, b* values and for Hue and Chroma.
(DOCX)

S4 Table Cololorimetric measurements of control and CAP treated samples inoculated with *L*. *monocytogenes*. Results represent the mean ± the standard error for L*, a*, b* values and for Hue and Chroma.
(DOCX)

S5 Table Overview of the total bacterial counts expressed as lg values for each experiment. Results represent the lg values and the mean ± standard error.
(DOCX)
