# Supplementary material for: Inactivation of Salmonella Typhimurium and Listeria monocytogenes on ham with nonthermal atmospheric pressure plasma
Source: PLoS One. 2018 May 24;13(5):e0197773. doi: 10.1371/journal.pone.0197773 (PMC5967798; doi:10.1371/journal.pone.0197773)
Supplement: S1 Table — Results represent the mean ± the standard error for L*, a*, b* values and for Hue and Chroma. (DOCX) [file pone.0197773.s001.docx]

| **Plasma Setup I (6.4 kV, 10 kHz, wet)** | | | | | | | | | | | | | | | | | | | | **Plasma Setup II (6.4 kV, 10 kHz, dry)** | | | | | | | | | | | | |  | |
| --- | --- | --- | --- | --- | --- | --- | --- | --- | --- | --- | --- | --- | --- | --- | --- | --- | --- | --- | --- | --- | --- | --- | --- | --- | --- | --- | --- | --- | --- | --- | --- | --- | --- | --- |
|  | | Untreated control | | | After storage | | | | Untreated control | | | After 10 min treatment | | | Untreated control | | | After 20 min treatment | | Untreated control | | | After storage | | | | Untreated control | | | After 10 min treatment | | Untreated control | | After 20 min treatment |
|  | | Initial values at day 0 | | | | | | | | | | | | | | | | | |  | | | | | | | | | | | | |  | |
| L* | | 56.38±3.05 | | - | | | | 53.21±0.68 | | | 54.96±2.56 | | | 54.18±1.67 | | | 54.69±4.46 | | | 56.75±2.53 | | - | | | | 56.21±1.86 | | | 56.93±2.26 | | 53.35±4.56 | | | 54.17±5.49 |
| a* | | 21.08±2.14 | | - | | | | 24.52±0.79 | | | 18.01±3.97 | | | 23.02±2.15 | | | 17.80±3.11 | | | 23.86±2.47 | | - | | | | 20.9±2.02 | | | 19.92±1.72 | | 21.35±3.03 | | | 20.07±2.41 |
| b* | | 15.38±1.48 | | - | | | | 16.90±0.19 | | | 13.66±0.19 | | | 16.66±0.88 | | | 13.70±3.09 | | | 16.64±0.66 | | - | | | | 14.93±0.89 | | | 15.16±1.15 | | 14.16±0.83 | | | 14.60±1.52 |
| Chroma | | 26.11±2.41 | | - | | | | 29.76±0.77 | | | 22.63±4.98 | | | 23.22±4.78 | | | 26.28±2.01 | | | 29.12±2.15 | | - | | | | 25.70±2.02 | | | 25.05±1.92 | | 25.68±2.52 | | | 24.90±2.05 |
| Hue | | 36.13±2.11 | | - | | | | 34.58±0.63 | | | 37.06±3.07 | | | 36.42±4.88 | | | 38.87±1.53 | | | 35.05±2.69 | | - | | | | 35.64±2 | | | 37.30±1.75 | | 33.94±4.37 | | | 36.19±4.66 |
| ΔE | | - | | | | | | 2.60±0.50 | | | | | | 2.66±0.49 | | | | | | - | | | | | | 1.84±0.95 | | | | | 2.53±0.70 | | | |
|  | | After 7 days of storage under MAP conditions | | | | | | | | | | | | | | | | |  |  | | | | | | | | | | | | | | |
| L* | 57.41±3.44 | | 59±2.90 | | | 55.69±3.86 | | | | 57.52±2.94 | | | 56.33±2.73 | | | 57.68±3.1 | | | | 58.87±3.38 | 59.04±4.33 | | | 56.20±2.96 | | | | 58.54±3.15 | | | 56.90±2.95 | | | 59.39±3.16 |
| a* | 20.04±3.08 | | 19.70±2.79 | | | 18.05±1.81 | | | | 19.26±2.02 | | | 19.05±2.26 | | | 19.71±2.30 | | | | 21.67±3.45 | 21.74±2.80 | | | 20.68±3.61 | | | | 22.03±3.68 | | | 19.52±2.56 | | | 21.62±2.84 |
| b* | 15.19±2.18 | | 16.56±1.04 | | | 14.24±2.52 | | | | 16.52±1.43 | | | 14.35±1.83 | | | 16.63±1.69 | | | | 16.36±1.01 | 17.08±1.03 | | | 14.91±1.17 | | | | 17.19±0.76 | | | 14.92±1.18 | | | 17.39±0.69 |
| Chroma | 25.33±3.75 | | 25.05±3.27 | | | 23.47±2.06 | | | | 25.43±2.11 | | | 24.32±3.04 | | | 25.80±3.04 | | | | 27.18±3.30 | 27.48±2.91 | | | 25.53±3.46 | | | | 28.02±3.13 | | | 24.61±2.41 | | | 27.78±2.48 |
| Hue | 37±2.75 | | 40.28±2.91 | | | 38.01±4.31 | | | | 40.5±2.70 | | | 38.29±2.51 | | | 40.22±2.09 | | | | 37.35±2.89 | 38.38±2.78 | | | 36.16±3.53 | | | | 38.39±4.34 | | | 37.58±3.32 | | | 39.05±3.15 |
| ΔE | 2.85±1.59 | | | | | 3.29±1.73 | | | | | | | 2.66±1.1 | | | | | | | 1.53±0.63 | | | | | 4.05±0.91 | | | | | | 4.26±1.42 | | | |
|  | After 14 days of storage under MAP conditions | | | | | | | | | | | | | | | | | |  |  | | | | | | | | | | | | | | |
| L* | 58.70±2.73 | | 59.6±2.77 | | | 56.81±3.25 | | | | 58.33±2.31 | | | 55.40±2.35 | | | 56.49±2.35 | | | | 56.62±2.98 | 56.66±3.03 | | | 55.68±4.88 | | | | 57.21±5.08 | | | 52.02±3.53 | | | 53.97±3.76 |
| a* | 19.89±2 | | 19.13±2.38 | | | 19.85±2.40 | | | | 20.32±3.53 | | | 19.05±2.51 | | | 19.96±2.67 | | | | 24.36±3.28 | 24.06±3.62 | | | 21.18±4.82 | | | | 22.05±5.48 | | | 24.50±4.02 | | | 25.69±3.63 |
| b* | 15.46±1.97 | | 16.11±1.79 | | | 15±1.61 | | | | 16.18±1.89 | | | 15.1±2.29 | | | 15.96±1.81 | | | | 16.95±0.85 | 17.66±1.05 | | | 15.46±1.10 | | | | 17.47±1.26 | | | 15.88±1.06 | | | 18.18±0.98 |
| Chroma | 25.21±2.61 | | 25.03±2.8 | | | 24.91±2.62 | | | | 26±3.83 | | | 23.86±3.03 | | | 25.54±3.22 | | | | 29.72±2.95 | 29.92±3.01 | | | 26.30±4.42 | | | | 28.23±5.04 | | | 29.37±3.63 | | | 31.55±3.10 |
| Hue | 37.80±2.38 | | 40.14±2.28 | | | 37.13±2.84 | | | | 38.78±2.63 | | | 37.14±1.81 | | | 38.65±1.33 | | | | 35.10±3.32 | 36.63±4.43 | | | 36.83±4.64 | | | | 39.17±4.89 | | | 33.40±4.38 | | | 35.58±3.98 |
| ΔE | 2.24±1.22 | | | | | | 2.77±1.47 | | | | | | 2.39±1.12 | | | | | | | 1.85±0.75 | | | | | 3.19±1.69 | | | | | | 3.64±1.49 | | | |
