# Supplementary material for: Inactivation of Salmonella Typhimurium and Listeria monocytogenes on ham with nonthermal atmospheric pressure plasma
Source: PLoS One. 2018 May 24;13(5):e0197773. doi: 10.1371/journal.pone.0197773 (PMC5967798; doi:10.1371/journal.pone.0197773)
Supplement: S2 Table — Results represent the mean ± the standard error for L*, a*, b* values and for Hue and Chroma. (DOCX) [file pone.0197773.s002.docx]

| **Plasma Setup III (10 kV, 2 kHz, wet)** | | | | | | | | | | | | | | | | | | | | **Plasma Setup IV (10 kV, 2 kHz, dry)** | | | | | | | | | | | | | |  | |
| --- | --- | --- | --- | --- | --- | --- | --- | --- | --- | --- | --- | --- | --- | --- | --- | --- | --- | --- | --- | --- | --- | --- | --- | --- | --- | --- | --- | --- | --- | --- | --- | --- | --- | --- | --- |
|  | | Untreated control | | | After storage | | | | Untreated control | | | After 10 min treatment | | | Untreated control | | | After 20 min treatment | | Untreated control | | | After storage | | | | | Untreated control | | | After 10 min treatment | | Untreated control | | After 20 min treatment |
|  | | Initial values at day 0 | | | | | | | | | | | | | | | | | |  | | | | | | | | | | | | | |  | |
| L* | | 55.75±0.64 | | - | | | | 53.13±1.50 | | | 53.31±1.06 | | | 56.06±3.18 | | | 56.44±2.36 | | | 57.05±2.41 | | - | | | | 54.80±1.67 | | | | 55.21±1.43 | | 54.68±2.33 | | | 55.06±2.79 |
| a* | | 26.76±0.97 | | - | | | | 25.11±1.32 | | | 23.99±0.69 | | | 21.50±3.06 | | | 19.58±2.44 | | | 23.86±2.22 | | - | | | | 21.68±1.1 | | | | 20.13±1.34 | | 21.26±2.83 | | | 19.21±1.85 |
| b* | | 18.17±0.72 | | - | | | | 16.63±1.22 | | | 16.48±1.36 | | | 16.14±0.88 | | | 15.96±1.15 | | | 16.88±0.95 | | - | | | | 15.6±1.28 | | | | 15.52±1.42 | | 14.85±0.94 | | | 15.04±0.68 |
| Chroma | | 32.34±0.73 | | - | | | | 30.14±1.22 | | | 29.12±0.69 | | | 26.84±2.89 | | | 25.39±2.44 | | | 29.27±1.54 | | - | | | | 26.75±0.70 | | | | 25.46±1.22 | | 25.95±2.75 | | | 24.43±1.43 |
| Hue | | 34.19±1.74 | | - | | | | 33.57±2.55 | | | 34.46±2.66 | | | 37.11±2.34 | | | 39.31±1.78 | | | 35.42±3.64 | | - | | | | 35.75±3.27 | | | | 37.63±3.41 | | 35.12±2.58 | | | 38.18±3.23 |
| ΔE | | - | | | | | | 1.38±0.74 | | | | | | 2.31±0.86 | | | | | | - | | | | | | | 1.88±0.53 | | | | | 2.49±1.55 | | | |
|  | | After 7 days of storage under MAP conditions | | | | | | | | | | | | | | | | |  |  | | | | | | | | | | | | | | | |
| L* | 58.06±2.28 | | 58.92±2.29 | | | 57.42±4.12 | | | | 59.84±2.98 | | | 53.71±0.63 | | | 56.23±1.51 | | | | 58.1±3.01 | 58.63±3.01 | | | 58.11±0.98 | | | | | 61.02±1.01 | | | 55.87±1.49 | | | 58.43±2.48 |
| a* | 23.62±2.53 | | 22.46±1.89 | | | 21.23±2.76 | | | | 21.55±2.80 | | | 23.39±0.67 | | | 23.16±0.80 | | | | 20.77±3.89 | 20.30±4.30 | | | 18.87±1.46 | | | | | 20.05±1.34 | | | 20.88±1.81 | | | 21.42±1.38 |
| b* | 16.96±0.62 | | 18.13±1.20 | | | 16.16±0.73 | | | | 18.08±1.11 | | | 15.89±1.20 | | | 17.68±1.19 | | | | 15.60±2.08 | 16.11±1.79 | | | 14.48±.94 | | | | | 17.43±0.38 | | | 15.19±0.65 | | | 17.16±0.44 |
| Chroma | 29.18±2.20 | | 28.87±2.20 | | | 26.70±2.60 | | | | 28.15±2.75 | | | 28.28±1.09 | | | 29.14±1.24 | | | | 26±4.27 | 25.95±4.41 | | | 23.79±1.67 | | | | | 26.57±1.25 | | | 25.84±1.67 | | | 27.46±1.09 |
| Hue | 36.05±2.58 | | 38.95±0.82 | | | 37.47±2.51 | | | | 40.16±2.48 | | | 34.16±1.67 | | | 37.32±1.40 | | | | 37.12±2.30 | 38.85±3.24 | | | 37.52±1.17 | | | | | 41.06±1.35 | | | 36.13±2.11 | | | 38.75±2.01 |
| ΔE | 2.21±1.47 | | | | | 3.40±0.98 | | | | | | | 3.19±1 | | | | | | | 1.51±0.39 | | | | | 4.40±0.47 | | | | | | | 3.50±1.44 | | | |
|  | After 14 days of storage under MAP conditions | | | | | | | | | | | | | | | | | |  |  | | | | | | | | | | | | | | | |
| L* | 55.91±1.61 | | 56.74±2.08 | | | 54.56±0.72 | | | | 56.92±0.87 | | | 55.99±2.69 | | | 58.11±2.94 | | | | 55.73±1.77 | 56.58±1.26 | | | 55.55±1.94 | | | | | 57.83±1.91 | | | 57.74±2.21 | | | 60.86±1.99 |
| a* | 25.37±1.83 | | 22.92±2.83 | | | 23.1±1 | | | | 23.46±1.29 | | | 21.46±3.21 | | | 21.93±2.30 | | | | 23.64±1.45 | 23.87±1 | | | 21.03±1.75 | | | | | 22.68±2.14 | | | 18.71±3 | | | 20.05±1.72 |
| b* | 17.24±0.86 | | 18.37±1.09 | | | 16.33±1.45 | | | | 18.52±0.98 | | | 16.47±1.08 | | | 18.78±0.78 | | | | 16.31±0.75 | 17.45±0.48 | | | 14.74±0.99 | | | | | 18.05±0.97 | | | 14.01±1.74 | | | 17.64±0.99 |
| Chroma | 30.70±1.68 | | 29.39±2.83 | | | 28.30±1.64 | | | | 29.89±1.60 | | | 27.08±3.05 | | | 28.89±2.1 | | | | 28.75±1.38 | 29.58±0.89 | | | 25.69±1.93 | | | | | 29.01±1.96 | | | 23.44±3.29 | | | 26.71±1.87 |
| Hue | 34.25±2.12 | | 38.87±2.09 | | | 35.21±1.30 | | | | 38.28±0.53 | | | 37.75±3.09 | | | 40.69±2.37 | | | | 34.63±1.60 | 36.19±1.25 | | | 35.07±1.27 | | | | | 38.6±2.56 | | | 37±2.39 | | | 41.41±1.44 |
| ΔE | 3.69±1.56 | | | | | | 3.20±0.84 | | | | | | 3.41±0.61 | | | | | | | 2.40±0.73 | | | | | 4.52±0.93 | | | | | | | 5.24±1.60 | | | |
