# Supplementary material for: Inactivation of Salmonella Typhimurium and Listeria monocytogenes on ham with nonthermal atmospheric pressure plasma
Source: PLoS One. 2018 May 24;13(5):e0197773. doi: 10.1371/journal.pone.0197773 (PMC5967798; doi:10.1371/journal.pone.0197773)
Supplement: S3 Table — Results represent the mean ± the standard error for L*, a*, b* values and for Hue and Chroma. (DOCX) [file pone.0197773.s003.docx]

| **Plasma Setup I (6.4 kV, 10 kHz, wet)** | | | | | | | | | | | | | | | | | | | | **Plasma Setup II (6.4 kV, 10 kHz, dry)** | | | | | | | | | | | | | |  | |
| --- | --- | --- | --- | --- | --- | --- | --- | --- | --- | --- | --- | --- | --- | --- | --- | --- | --- | --- | --- | --- | --- | --- | --- | --- | --- | --- | --- | --- | --- | --- | --- | --- | --- | --- | --- |
|  | | Untreated control | | | After storage | | | | Untreated control | | | After 10 min treatment | | | Untreated control | | | After 20 min treatment | | Untreated control | | | After storage | | | | | Untreated control | | | After 10 min treatment | | Untreated control | | After 20 min treatment |
|  | | Initial values at day 0 | | | | | | | | | | | | | | | | | |  | | | | | | | | | | | | | |  | |
| L* | | 56.95±2.31 | | - | | | | 55±1.62 | | | 55.57±2.37 | | | 56.62±2.83 | | | 57.75±3.38 | | | 53.37±4.50 | | - | | | | 52.60±5.54 | | | | 53.22±6.54 | | 53.6±2.84 | | | 54.20±4.12 |
| a* | | 23.63±1.48 | | - | | | | 22.66±1.73 | | | 21.82±1.77 | | | 20.24±2.31 | | | 19.67±1.47 | | | 23.54±2.03 | | - | | | | 20.83±2.50 | | | | 20.31±1.94 | | 19.72±1.85 | | | 19.22±3.64 |
| b* | | 16.78±65 | | - | | | | 15.75±0.81 | | | 15.96±1.38 | | | 15.10±0.52 | | | 15.93±1.03 | | | 14.89±2.09 | | - | | | | 13.40±1.20 | | | | 14.07±1.76 | | 13.07±1.76 | | | 14.09±2.56 |
| Chroma | | 28.99±1.41 | | - | | | | 27.52±1.75 | | | 26.79±2.26 | | | 25.29±1.94 | | | 25.34±1.20 | | | 27.91±2.27 | | - | | | | 24.79±1.94 | | | | 24.82±1.69 | | 23.68±2.31 | | | 23.85±4.12 |
| Hue | | 37.74±1.53 | | - | | | | 34.93±1.82 | | | 36.19±2.18 | | | 36.99±3.04 | | | 39.06±3.02 | | | 32.38±3.81 | | - | | | | 32.97±4.48 | | | | 34.76±4.70 | | 33.56±2.71 | | | 36.23±2.27 |
| ΔE | | - | | | | | | 2.21±0.82 | | | | | | 2.42±1.02 | | | | | | - | | | | | | | 2.19±1.14 | | | | | 2.93±1.06 | | | |
|  | | After 7 days of storage under MAP conditions | | | | | | | | | | | | | | | | |  |  | | | | | | | | | | | | | | | |
| L* | 59.58±2.36 | | 59.96±1.85 | | | 55.38±2.65 | | | | 57.89±2.48 | | | 57.74±3.19 | | | 60.17±3.24 | | | | 54.70±2.60 | 55.66±2.31 | | | 53.63±1.97 | | | | | 56.40±2.25 | | | 55.84±3.75 | | | 58.20±3.43 |
| a* | 21.63±2.79 | | 21.49±2.47 | | | 22.17±2.42 | | | | 22.01±2.88 | | | 19.31±3.48 | | | 19.26±3.44 | | | | 25.65±2.60 | 24.24±1.66 | | | 22.87±2.15 | | | | | 22.21±0.77 | | | 21.64±3.62 | | | 22.28±2.06 |
| b* | 16.67±0.65 | | 16.76±0.74 | | | 15.82±0.83 | | | | 17.62±0.86 | | | 15.75±1.11 | | | 17.16±0.54 | | | | 17.20±1.32 | 17.61±1.0 | | | 15.74±1.48 | | | | | 18.08±0.83 | | | 14.25±1.49 | | | 17.20±1.28 |
| Chroma | 27.34±2.4 | | 27.30±2.03 | | | 27.87±2 | | | | 28.26±2.49 | | | 24.98±3.14 | | | 25.56±2.73 | | | | 30.34±2.33 | 29.88±1.78 | | | 27.83±2.53 | | | | | 29.43±0.84 | | | 25.99±3.36 | | | 28.17±2.04 |
| Hue | 37.85±3.17 | | 38.15±3.33 | | | 35.71±3.47 | | | | 38.93±3.48 | | | 39.63±4.31 | | | 42.09±4.77 | | | | 34.20±2.03 | 36.02±1.92 | | | 34.70±1.97 | | | | | 37.92±1.49 | | | 33.67±4.43 | | | 37.74±2.59 |
| ΔE | 1.50±0.57 | | | | | 3.75±0.87 | | | | | | | 3.49±1.28 | | | | | | | 1.76±0.91 | | | | | 4.13±1.05 | | | | | | | 4.33±0.91 | | | |
|  | After 14 days of storage under MAP conditions | | | | | | | | | | | | | | | | | |  |  | | | | | | | | | | | | | | | |
| L* | 57.21±2.27 | | 57.90±2.12 | | | 55.98±1.54 | | | | 58.36±1.74 | | | 56.09±2.95 | | | 57.86±2.95 | | | | 53.32±2.70 | 53.94±2.7 | | | 51.62±2.82 | | | | | 52.55±2.63 | | | 52.98±2.46 | | | 56.92±2.55 |
| a* | 24.28±2.52 | | 23.47±2.37 | | | 21.33±1.66 | | | | 22.02±1.22 | | | 20.87±3.35 | | | 21.39±2.96 | | | | 27.87±2.85 | 26.80±2.60 | | | 24.94±2.53 | | | | | 27.10±1.59 | | | 24.07±2.61 | | | 24.24±2.51 |
| b* | 17.06±0.46 | | 17.37±0.48 | | | 15.57±0.57 | | | | 17.53±0.46 | | | 15.57±0.97 | | | 17.38±0.97 | | | | 17.50±0.88 | 18.58±0.69 | | | 15.11±0.94 | | | | | 17.80±0.49 | | | 15.33±1.36 | | | 18.14±0.93 |
| Chroma | 29.71±2.11 | | 29.23±1.96 | | | 26.44±1.57 | | | | 28.15±1.15 | | | 26.08±3.14 | | | 27.60±2.81 | | | | 32.93±2.81 | 32.64±2.37 | | | 29.19±2.34 | | | | | 31.95±1.99 | | | 28.56±2.64 | | | 30.33±1.98 |
| Hue | 35.32±2.88 | | 36.66±2.86 | | | 36.20±1.70 | | | | 38.55±1.29 | | | 37.10±3.56 | | | 39.35±2.94 | | | | 32.25±1.89 | 34.87±2.32 | | | 31.35±2.85 | | | | | 33.44±1.36 | | | 32.60±2.17 | | | 36.98±3.51 |
| ΔE | 1.57±0.73 | | | | | | 3.32±0.72 | | | | | | 2.96±0.76 | | | | | | | 1.96±0.60 | | | | | 3.82±1.21 | | | | | | | 4.70±1.93 | | | |
