# Supplementary material for: Inactivation of Salmonella Typhimurium and Listeria monocytogenes on ham with nonthermal atmospheric pressure plasma
Source: PLoS One. 2018 May 24;13(5):e0197773. doi: 10.1371/journal.pone.0197773 (PMC5967798; doi:10.1371/journal.pone.0197773)
Supplement: S4 Table — Results represent the mean ± the standard error for L*, a*, b* values and for Hue and Chroma. (DOCX) [file pone.0197773.s004.docx]

| **Plasma Setup III (10 kV, 2 kHz, wet)** | | | | | | | | | | | | | | | | | | | | **Plasma Setup IV (10 kV, 2 kHz, dry)** | | | | | | | | | | | | | |  | |
| --- | --- | --- | --- | --- | --- | --- | --- | --- | --- | --- | --- | --- | --- | --- | --- | --- | --- | --- | --- | --- | --- | --- | --- | --- | --- | --- | --- | --- | --- | --- | --- | --- | --- | --- | --- |
|  | | Untreated control | | | After storage | | | | Untreated control | | | After 10 min treatment | | | Untreated control | | | After 20 min treatment | | Untreated control | | | After storage | | | | | Untreated control | | | After 10 min treatment | | Untreated control | | After 20 min treatment |
|  | | Initial values at day 0 | | | | | | | | | | | | | | | | | |  | | | | | | | | | | | | | |  | |
| L* | | 58.87±2.05 | | - | | | | 56.37±2.55 | | | 56.51±2.71 | | | 55.86±1.54 | | | 56.22±1.78 | | | 54.86±1.39 | | - | | | | 54.778±1.54 | | | | 55.96±0.63 | | 54.23±1.81 | | | 53.86±2.05 |
| a* | | 22.51±2,55 | | - | | | | 21.30±2.36 | | | 19.39±2.31 | | | 21.70±2.03 | | | 19.80±1.88 | | | 24.82±0.98 | | - | | | | 22.08±1.77 | | | | 20.22±1.13 | | 23.29±1.73 | | | 21.05±1.53 |
| b* | | 17.19±0.56 | | - | | | | 15.56±0.53 | | | 15.35±0.65 | | | 15.78±0.64 | | | 15.86±1.13 | | | 16.86±0.52 | | - | | | | 15.20±1.22 | | | | 15.13±0.66 | | 16.14±1.14 | | | 15.88±1.20 |
| Chroma | | 28.35±2.27 | | - | | | | 26.40±2.09 | | | 24.77±1.98 | | | 26.95±1.51 | | | 25.41±1.25 | | | 29.99±1.10 | | - | | | | 26.90±1.86 | | | | 25.25±1.25 | | 28.34±2.00 | | | 26.39±1.58 |
| Hue | | 37.54±2.62 | | - | | | | 36.31±2.64 | | | 38.54±2.64 | | | 36.08±3.48 | | | 38.84±4.02 | | | 34.20±0.50 | | - | | | | 34.56±1.73 | | | | 36.82±0.86 | | 34.74±1.16 | | | 37.10±2.45 |
| ΔE | | - | | | | | | 2.08±0.28 | | | | | | 2.23±0.36 | | | | | | - | | | | | | | 2.91±1.50 | | | | | 3±1.44 | | | |
|  | | After 7 days of storage under MAP conditions | | | | | | | | | | | | | | | | |  |  | | | | | | | | | | | | | | | |
| L* | 57.82±1.72 | | 56.25±0.90 | | | 53.56±2.61 | | | | 55.61±2.54 | | | 53.61±2.52 | | | 55.14±3.34 | | | | 53.87±2.36 | 54.75±1.62 | | | 54.38±1.27 | | | | | 54.91±2.20 | | | 53.67±0.79 | | | 54.82±2.05 |
| a* | 23.48±1.98 | | 23.93±1.03 | | | 24.22±2.54 | | | | 25.85±1.80 | | | 24.17±2.03 | | | 25.31±3.16 | | | | 25.96±3.53 | 23.86±3.12 | | | 22.43±0.90 | | | | | 22.82±2.69 | | | 23.98±1.29 | | | 23.46±1.85 |
| b* | 17.87±0.33 | | 17.80±0.59 | | | 16.11±0.63 | | | | 17.92±0.50 | | | 15.85±0.54 | | | 18.02±0.97 | | | | 17.10±0.74 | 17.35±0.41 | | | 16.38±1.35 | | | | | 17.16±1.85 | | | 15.44±0.81 | | | 17.53±0.57 |
| Chroma | 29.54±1.42 | | 29.84±0.76 | | | 29.11±2.23 | | | | 30.82±1.74 | | | 28.94±1.83 | | | 31.09±2.98 | | | | 31.11±3.31 | 29.55±2.57 | | | 27.80±1.13 | | | | | 28.57±3.07 | | | 28.54±0.88 | | | 29.30±1.59 |
| Hue | 37.38±2.76 | | 36.67±1.74 | | | 33.77±2.67 | | | | 35.63±1.82 | | | 33.34±1.98 | | | 35.63±2.70 | | | | 33.59±2.62 | 36.28±3.62 | | | 36.11±2,42 | | | | | 36.99±2.23 | | | 32.82±2.52 | | | 36.84±2.18 |
| ΔE | 2.26±1.13 | | | | | 3.09±0.70 | | | | | | | 3.23±1.54 | | | | | | | 2.57±1.51 | | | | | 3.35±1.70 | | | | | | | 3.62±0.41 | | | |
|  | After 14 days of storage under MAP conditions | | | | | | | | | | | | | | | | | |  |  | | | | | | | | | | | | | | | |
| L* | 57.55±1.18 | | 56.51±0.86 | | | 52.88±2.48 | | | | 55.28±2.73 | | | 52.38±2.02 | | | 54.39±2.70 | | | | 55.13±1.25 | 54.85±1.46 | | | 52.18±1.74 | | | | | 54.88±2.49 | | | 55.12±1.54 | | | 57.69±1.71 |
| a* | 23.60±1.23 | | 23.74±0.71 | | | 23.87±1.79 | | | | 24.89±2.18 | | | 24.65±3 | | | 24.59±3.10 | | | | 26.24±2.06 | 23.29±3.45 | | | 24.93±2.55 | | | | | 23.50±2.63 | | | 22.51±0.73 | | | 20.67±1.80 |
| b* | 17.58±1.01 | | 17.95±0.40 | | | 15.18±0.78 | | | | 17.56±0.94 | | | 15.70±0.70 | | | 17.92±0.92 | | | | 17.57±0.38 | 17.56±0.71 | | | 16.01±0.76 | | | | | 18.02±0.82 | | | 15.83±0.67 | | | 18.55±0.79 |
| Chroma | 29.46±0.53 | | 29.77±0.69 | | | 28.30±1.84 | | | | 30.48±2.23 | | | 29.24±2.87 | | | 30.45±2.88 | | | | 31.60±1.61 | 29.22±2.99 | | | 29.61±2.22 | | | | | 29.66±2.14 | | | 27.58±0.92 | | | 27.80±1.40 |
| Hue | 36.77±2.89 | | 37.11±0.82 | | | 32.50±1.34 | | | | 35.27±1.59 | | | 32.66±2.25 | | | 36.27±2.72 | | | | 33.91±2.45 | 37.33±3.86 | | | 32.86±2.97 | | | | | 37.66±3.41 | | | 35.45±0.68 | | | 41.98±2.90 |
| ΔE | 2.04±0.94 | | | | | | 4.15±0.88 | | | | | | 3.25±1.38 | | | | | | | 2.57±1.22 | | | | | 3.97±0.86 | | | | | | | 4.55±1.56 | | | |
