# Supplementary material for: Inactivation of Salmonella Typhimurium and Listeria monocytogenes on ham with nonthermal atmospheric pressure plasma
Source: PLoS One. 2018 May 24;13(5):e0197773. doi: 10.1371/journal.pone.0197773 (PMC5967798; doi:10.1371/journal.pone.0197773)
Supplement: S5 Table — Results represent the lg values and the mean ± standard error. (DOCX) [file pone.0197773.s005.docx]

|  | *S.* Typhimurium | | | | *L. monocytogenes* | | | | | | |
| --- | --- | --- | --- | --- | --- | --- | --- | --- | --- | --- | --- |
| Plasma-setup | 1 (lg CFU/g) | 2 (lg CFU/g) | 3 (lg CFU/g) | 4 (lg CFU/g) | 1 (lg CFU/g) | 2 (lg CFU/g) | | 3 (lg CFU/g) | | 4 (lg CFU/g) | |
| 0min/0 days storage | 5.30 ± 0.49 | 4.46 ± 0.73 | 4.71 ± 0.12 | 4.69 ± 0.20 | 3.90 ± 0.28 | | 3.49 ± 0.14 | | 4.07 ± 0.09 | | 4.02 ± 0.18 |
| 10 min/0 days storage | 4.93 ± 0.56 | 4.14 ± 0.17 | 4.12 ± 0.17 | 4.01 ± 0.38 | 3.62 ± 0.28 | | 3.17 ± 0.21 | | 3.52 ± 0.11 | | 3.43 ± 0.33 |
| 20 min/0 days storage | 4.72 ± 0.56 | 3.81 ± 0.25 | 3.57 ± 0.12 | 3.73 ± 0.22 | 3.44 ± 0.37 | | 2.81 ± 0.22 | | 3.16 ± 0.20 | | 3 ± 0.32 |
| 0 min/7 days storage | 5.38 ± 0.58 | 4.72 ± 0.26 | 4.82 ± 0.06 | 4.74 ± 0.13 | 3.79 ± 0.31 | | 3.36 ± 0.13 | | 4.01 ± 0.03 | | 3.95 ± 0.08 |
| 10 min/7 days storage | 4.79 ± 0.51 | 3.75 ± 0.18 | 3.84 ± 0.11 | 3.88 ± 0.18 | 3.40 ± 0.32 | | 2.97 ± 0.19 | | 3.04 ± 0.49 | | 3.1 ± 0.44 |
| 20 min/7 days storage | 4.40 ± 0.63 | 3.61 ± 0.23 | 3.38 ± 0.13 | 3.44 ± 0.25 | 3.24 ± 0.28 | | 2.71 ± 0.34 | | 2.70 ± 0.61 | | 2.42 ± 0.20 |
| 0 min/14 days storage | 5.25 ± 0.44 | 4.48 ± 0.24 | 4.79 ± 0.07 | 4.57 ± 0.17 | 3.80 ± 0.37 | | 3.59 ± 0.39 | | 4.01 ± 0.07 | | 3.93 ± 0.18 |
| 10 min/14 days storage | 4.56 ± 0.57 | 3.78 ± 0.18 | 3.51 ± 0.19 | 3.52 ± 0.31 | 3.31 ± 0.55 | | 2.25 ± 0.77 | | 2.23 ± 0.17 | | 2.42 ± 0.22 |
| 20 min/14 days storage | 4.43 ± 0.63 | 3.44 ± 0.28 | 2.86 ± 0.12 | 3.12 ± 0.52 | 2.97 ± 0.79 | | 1.81 ± 0.74 | | 1.72 ± 0.55 | | 1.46 ± 0.85 |
